# Human anti-CAIX antibodies mediate immune cell inhibition of renal cell carcinoma *in vitro* and in a humanized mouse model *in vivo*

**DOI:** 10.1186/s12943-015-0384-3

**Published:** 2015-06-11

**Authors:** De-Kuan Chang, Raymond J. Moniz, Zhongyao Xu, Jiusong Sun, Sabina Signoretti, Quan Zhu, Wayne A. Marasco

**Affiliations:** Department of Cancer Immunology and AIDS, Dana-Farber Cancer Institute, 450 Brookline Ave., Boston, MA USA; Department of Medicine, Harvard Medical School, 25 Shattuck Street, Boston, MA USA; Department of Medical Oncology, Dana-Farber Cancer Institute, 450 Brookline Ave., Boston, MA USA; Department of Pathology, Brigham and Women’s Hospital, Harvard Medical School, Boston, MA USA

**Keywords:** Orthotopic tumor, NSG mouse, Antibody engineering, Carbonic anhydrase IX, Immunotherapy, Xenograft

## Abstract

**Background:**

Carbonic anhydrase (CA) IX is a surface-expressed protein that is upregulated by the hypoxia inducible factor (HIF) and represents a prototypic tumor-associated antigen that is overexpressed on renal cell carcinoma (RCC). Therapeutic approaches targeting CAIX have focused on the development of CAIX inhibitors and specific immunotherapies including monoclonal antibodies (mAbs). However, current *in vivo* mouse models used to characterize the anti-tumor properties of fully human anti-CAIX mAbs have significant limitations since the role of human effector cells in tumor cell killing *in vivo* is not directly evaluated.

**Methods:**

The role of human anti-CAIX mAbs on CAIX^+^ RCC tumor cell killing by immunocytes or complement was tested *in vitro* by antibody-dependent cell-mediated cytotoxicity (ADCC), complement-dependent cytotoxicity (CDC) and antibody-dependent cellular phagocytosis (ADCP) as well as on CAIX^+^ RCC cellular motility, wound healing, migration and proliferation. The *in vivo* therapeutic activity mediated by anti-CAIX mAbs was determined by using a novel orthotopic RCC xenograft humanized animal model and analyzed by histology and FACS staining.

**Results:**

Our studies demonstrate the capacity of human anti-CAIX mAbs that inhibit CA enzymatic activity to result in immune-mediated killing of RCC, including nature killer (NK) cell-mediated ADCC, CDC, and macrophage-mediated ADCP. The killing activity correlated positively with the level of CAIX expression on RCC tumor cell lines. In addition, Fc engineering of anti-CAIX mAbs was shown to enhance the ADCC activity against RCC. We also demonstrate that these anti-CAIX mAbs inhibit migration of RCC cells *in vitro*. Finally, through the implementation of a novel orthotopic RCC model utilizing allogeneic human peripheral blood mononuclear cells in NOD/SCID/IL2Rγ^−/−^ mice, we show that anti-CAIX mAbs are capable of mediating human immune response *in vivo* including tumor infiltration of NK cells and activation of T cells, resulting in inhibition of CAIX^+^ tumor growth.

**Conclusions:**

Our findings demonstrate that these novel human anti-CAIX mAbs have therapeutic potential in the unmet medical need of targeted killing of HIF-driven CAIX^+^RCC. The orthotopic tumor xenografted humanized mouse provides an improved model to evaluate the *in vivo* anti-tumor capabilities of fully human mAbs for RCC therapy.

**Electronic supplementary material:**

The online version of this article (doi:10.1186/s12943-015-0384-3) contains supplementary material, which is available to authorized users.

## Introduction

Globally, an estimated 270,000 new cases and 116,000 deaths attributed to kidney cancer occur each year [1]. Greater than 90 % of kidney neoplasms are classified as renal cell carcinoma (RCC), of which upwards of 30 % progress towards metastatic disease [2, 3]. Treatment of RCC can be broadly grouped into either immunotherapy or targeted therapy. Immunotherapy with high dose interleukin-2 (IL-2) alone or in combination with interferon-α (IFN-α) has historically been a frontline of defense against RCC [4]. More recently, checkpoint control inhibitors and targeted therapies have shown great promise in combating RCC [2, 5, 6]. The currently approved targeted therapies against RCC include a monoclonal antibody (mAb) that blocks vascular endothelial growth factor from binding its receptor (bevacizumab), tyrosine kinase inhibitors (TKIs; sunitinib, sorafenib, pazopanib, and axitinib), and mammalian target of rapamycin inhibitors (everolimus and temsirolimus). These therapies alone or in combination have shown promise towards combating RCC [7, 8], but a complete and curative regimen by these drug or immunotherapy agents remains elusive.

Tumor-associated antigens are the focus of both diagnostic and therapeutic strategies against many forms of cancer, and carbonic anhydrase (CA) IX is the most well characterized antigen associated with RCC [9, 10]. CAIX is a trans-membrane protein that alters the pH of the extracellular microenvironment towards acidity during hypoxia [10–12]. Expression of CAIX is controlled by the tumor suppressor von Hippel-Lindau factor, and nearly all forms of clear cell RCC express CAIX, which is absent on normal kidney tissue [9]. Expression of CAIX on RCC is a prognostic factor, in that reduced CAIX expression is closely associated with the progression of disease [13] while expression of CAIX correlates with the efficacy of some treatments [4]. Thus, although expression of CAIX may provide RCC tumors with a growth advantage [14–16], it also conveys a level of susceptibility to treatment.

Multiple immunotherapeutic approaches center on targeting CAIX, including cellular-based strategies to vaccinate against CAIX [17–19] and mAbs that bind CAIX [20–23]. The chimeric antibody girentuximab (Rencarex®, Wilex) has been extensively tested in clinical trials [24], including its use alone, coupled to radioisotopes, in combination with cytokines, and in conjunction with chimeric antigen receptor (CAR) T cell therapy [25]. Results were recently released from a Phase 3 trial testing the efficacy of girentuximab alone versus placebo to limit disease progression in patients having undergone nephrectomy of non-metastatic clear cell RCC [26]. While the study objectives were not met, preliminary data indicate that a higher degree of efficacy was seen in younger patients whose tumors expressed high levels of CAIX. These findings, in conjunction with other clinical and preclinical data, emphasize the potential for anti-CAIX mAbs in treating RCC, but also highlight the need for new anti-CAIX mAbs that may have more potent anti-tumor properties [2, 27].

In seeking to develop novel therapeutic agents against RCC and other CAIX-expressing malignancies, we sought to further characterize a panel of high affinity, fully human antibodies against CAIX that we had previously described [23]. We focused on two high-affinity antibodies that inhibit CA activity, but have disparate capabilities to induce CAIX internalization. We examined their ability to elicit cellular immune responses to CAIX expressing tumor cells both *in vitro* and *in vivo*. Our results demonstrate that these two human anti-CAIX mAbs mediate immune killing of CAIX^+^ tumor cells *in vitro* and show potent therapeutic activity *in vivo*.

## Results

### Fully human anti-CAIX antibodies mediate ADCC *in vitro*

**Fig. 1:**
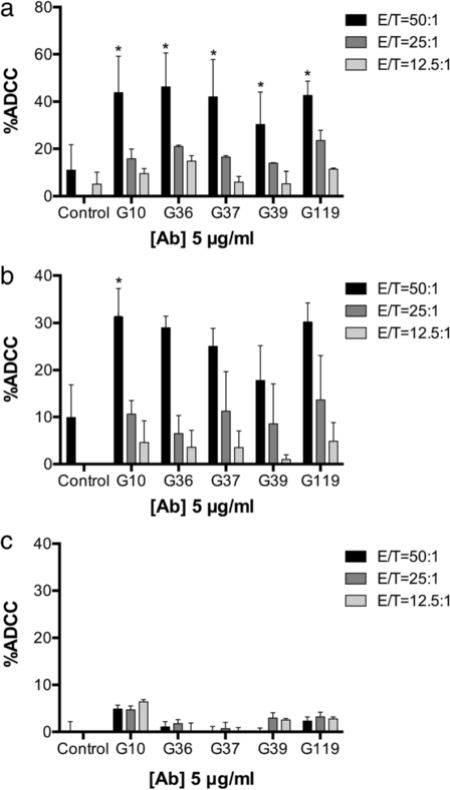
ADCC activity of anti-CAIX scFv-Fc antibodies. Anti-CAIX scFv-Fc antibodies G10, G36, G37, G39, and G119, were tested for ADCC activity against RCC cells with high CAIX expression (SKRC-09, **a**), moderate CAIX expression (SKRC-52, **b**), and low/negative CAIX expression (SKRC-59, **c**). Human PBMC were isolated from healthy donors, and total PBMC counted as effector cells (E) in co-culture with the respective target RCC cell line (T). The concentration of antibodies was 5 μg/ml. Culture supernatant after four hours of co-culture was examined for LDH as a measure of cytotoxicity. Data represent the mean of three independent experimental values, ± S.E.M each experiment performed in triplicate.. * represents p value of two-way ANOVA < 0.05, compared to control antibody

Our previous report defined a novel panel of fully human anti-CAIX antibodies that varied in their affinity, capacity to inhibit the enzymatic activity of CAIX, and ability to induce internalization of CAIX upon binding [23]. To ascertain the ability of these antibodies to mediate immune cell killing of CAIX-expressing RCC cells, we chose to analyze high affinity scFv-Fc antibodies that either inhibited the CA activity of CAIX, but did not internalize (G37 and G39), or showed minimal to modest inhibition of CA activity and induced internalization of CAIX (G10, G36, and G119) [23]. We utilized RCC cell lines with high CAIX (SKRC-09, Fig. 1a), moderate CAIX (SKRC-52, Fig. 1b), or no CAIX expression (SKRC-59, Fig. 1c), and found that all five antibodies mediate ADCC in accordance with the relative expression level of CAIX on RCC lines (Fig. 1 and Additional file 1: Figure S1A) [28]. Interestingly, the antibodies that induce internalization of CAIX (G10, G36, and G119) showed comparable ADCC activity to those that do not internalize (G37 and G39), suggesting that, upon binding to the CAIX epitope (s), the Fc regions of all antibodies from both groups are properly positioned to promote Fcγ receptor (FcγR)-mediated killing.

### Both internalizing and non-internalizing anti-CAIX antibodies limit RCC migration

**Fig. 2:**
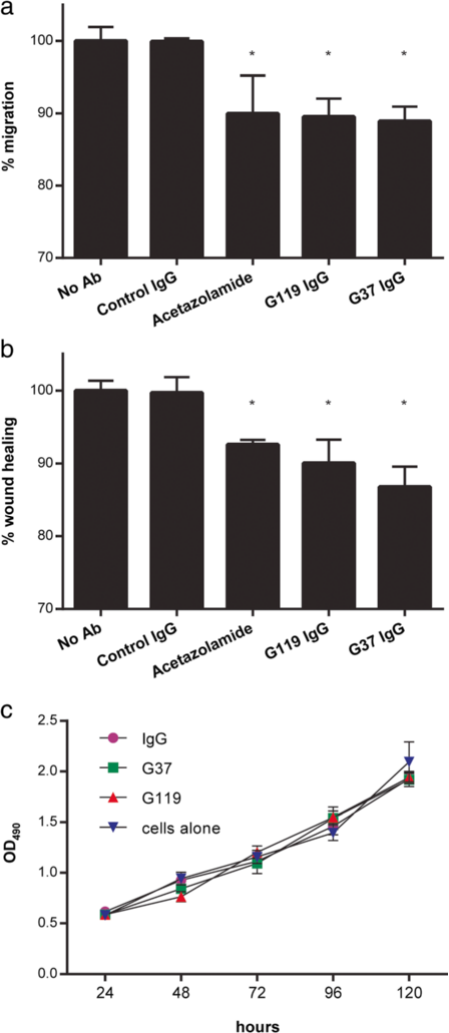
Anti-CAIX IgG1 mAbs modulate the motility of CAIX^+^ RCC. (**a**) Cell migration assayed by transwell migration, using CAIX^+^ SKRC-52 cells and treatment with anti-CAIX mAbs (2.5 μg/ml), non-specific control antibody (2.5 μg/ml), or acetazolamide (AZ, 100 μM). Migration was measured after 24 h, in response to HGF in the lower chamber. (**b**) Wound healing assay of CAIX^+^ RCC cells in the presence of anti-CAIX mAbs (10 μg/ml), non-specific control antibody (10 μg/ml), or acetazolamide (AZ, 100 μM). Confluent monolayer of SKRC-52 cells were wounded, and after 24 h healing calculated as the area not containing cells as measured by microscopy. (**c**) Cellular proliferation of CAIX^+^ RCC cells, measured in conditions as above, through 4 days post-treatment. All data represent mean values ± S.D. of three independent experiments, each experiment performed in triplicate. * represents p value of student *t*-test < 0.05, compared to control antibody

In light of observations that CAIX activity has been linked to the capacity of tumor cells to migrate and grow, we questioned whether antibodies that are capable of blocking CAIX activity could limit RCC migration *in vitro* [29]. Two full-length IgG1 anti-CAIX mAbs that exhibited a high (G37) or moderate (G119) capacity to block CA activity and were internalizing or not, respectively, were tested. In a transwell assay (Fig. 2a), both anti-CAIX mAbs G37 and G119 showed inhibition of RCC cell migration comparable to that seen with the CA inhibitor acetazolamide. Similarly, both mAbs showed a capacity to inhibit RCC growth in wound healing assays (Fig. 2b) that mirrored inhibition seen with acetazolamide treatment [29]. An isotype control IgG1 did not have these properties. Furthermore, cell proliferation remained unaltered in the presence of anti-CAIX mAbs in a MTT assay (Fig. 2c), suggesting that anti-CAIX mAbs do not directly affect RCC viability. Together, the data demonstrate that anti-CAIX G37 and G119 IgG1 mAbs are capable of inhibiting RCC migration.

### Anti-CAIX mAbs can be engineered to enhance ADCC effector function

**Fig. 3:**
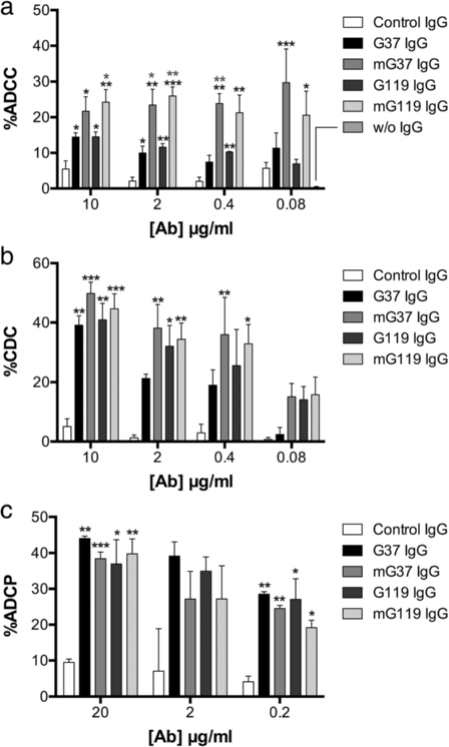
The antibody-mediated killing activities of wild type and Fc mutant G37 and G119 on CAIX expressing tumor cells. (**a**) Anti-CAIX mAbs (G37 and G119) and the variants (mG37 and mG119) were tested for ADCC activity with human PBMCs in a dose dependent manner. CAIX^+^ SKRC-52 cells were incubated with human PBMCs at a ratio of 1:25 and the indicated concentrations of antibodies, and cytotoxicity measured by LDH release in the culture supernatant. Data represent three independent experiments, each performed in triplicate. (**b**) CDC activity was determined by culture of rabbit serum with SKRC-52 cells, and cytotoxicity measured as above after 6 h. Data represent three independent experiments, each performed in triplicate. (**c**) ADCP activity was determined in vitro by flow cytometry, measuring the uptake of fluorescently labeled SKRC-52 cells (PKH) by monocyte derived human macrophages. Data represent the mean percentage of PKH^+^CD16^+^ macrophages from two experiments, as determined by FACS, each experiment performed in triplicate. All data points represent the mean value ± S.E.M.. *, **, and *** represent p value of two-way ANOVA < 0.05, 0.01, and 0.005, respectively (grey asterisks, compared to wild type IgG1; black asterisks, compared to control IgG1)

Several studies have demonstrated that mutations in the Fc region of IgG1 can enhance antibody affinity for FcγR in a manner that increases their effector activity [30, 31]. We engineered these mutations into the Fc region of G37 and G119 IgG1 by changing amino acids S239D/H268F/S324T/I332E (which do not only alter FcγR binding of IgG1 but also C1q binding) [31, 32], and then examined the capacity of these mutations to enhance effector function through ADCC, CDC and ADCP. In comparison to native IgG1 formats, the mutated forms of both G37 and G119 (mIgG1) demonstrated increased effector activity in ADCC assays (Fig. 3a). Both wild type G37 and G119 and their mIgG1 isoforms showed CDC and ADCP activity in a dose dependent fashion (Figs. 3b and c, and Additional file 1: Figure S1b). However, the mIgG1 isoforms while showing an increasing trend in killing, did not show statistically significant enhancement of CDC activity compared to wild type IgG1. In addition, the mIgG1 isoforms showed a small but not significantly lower ADCP activity compared to wild type IgG1. These experiments demonstrate that mutations in the Fc region of IgG1 can enhance the ADCC effector activity of the anti-CAIX mAbs *in vitro*.

### Anti-CAIX mAbs limit tumor growth in an orthotopic model of RCC

**Fig. 4:**
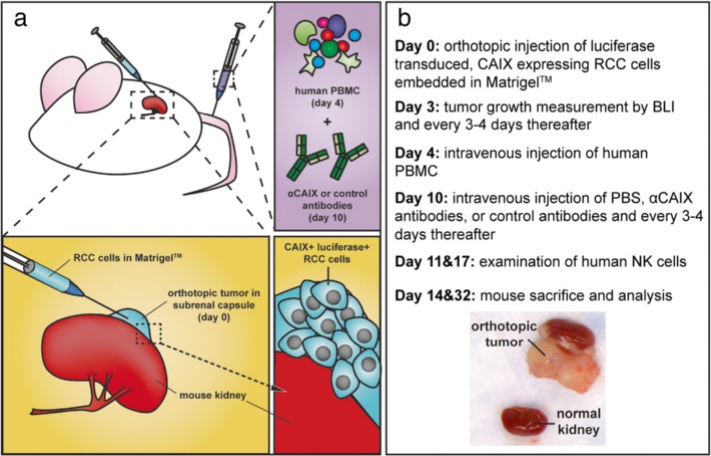
Design of the orthotopic RCC model and humanization with PBMC. (**a**) Diagram of the orthotopic implantation and engraftment of a human RCC line, transduced with luciferase, into the subrenal capsule of NSG mice. Subsequent to tumor engraftment, human PBMC and then anti-CAIX or control IgG1 treatment was administered to mice intravenously, with repeated dosing of treatment and measurement of tumor growth by BLI every 3–4 days. (**b**) Outline of establishment and treatment of the orthotopic model

The capacity of the anti-CAIX mAbs to elicit immune killing of RCC, and in turn their therapeutic potential, is best tested *in vivo*. While several reports have utilized xenograft models of RCC to assay the mechanisms and efficacy of targeted therapies [33], these models are limited in their capacity to assay the efficacy of antibody therapies that leverage human immune cell killing of engrafted tumors. To address this shortcoming, we developed a novel humanized mouse model that utilized allogeneic human peripheral blood mononucleated cells (PBMC) to mediate inhibition of orthotopic RCC tumor growth (Fig. 4). Orthotopic models offer a means to mimic clinically relevant tumor growth [5, 34] (Fig. 4a), which we hypothesized could be inhibited by treatment with human immune cells and subsequent administration of anti-CAIX mAbs (Fig. 4b). Utilizing this model, we tested wild type IgG1 and mIgG1 formats of anti-CAIX G37 and G119 and compared the ability of these mAbs to inhibit tumor growth versus PBS or a non-specific IgG1 control.

**Fig. 5:**
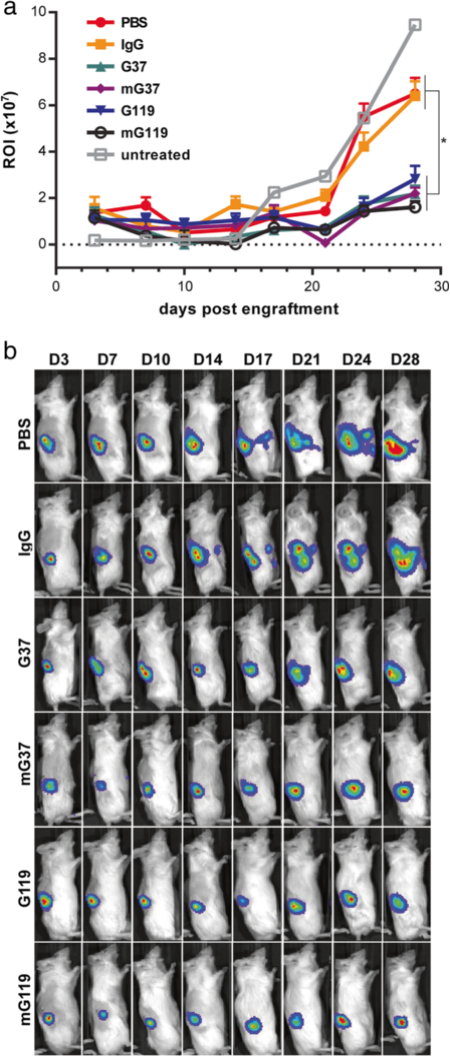
Orthotopic RCC tumor growth following treatment with anti-CAIX mAbs. (**a**) Tumor growth was determined in each treatment group by BLI, following injection of antibodies and human PBMC as described in Fig. 4. Data represent measurement of three mice and are representative of two independent experiments. * indicates p value of two-way ANOVA < 0.05, compared to control IgG. (**b**) Representative images of BLI measurement depicted in (**a**)

**Fig. 6:**
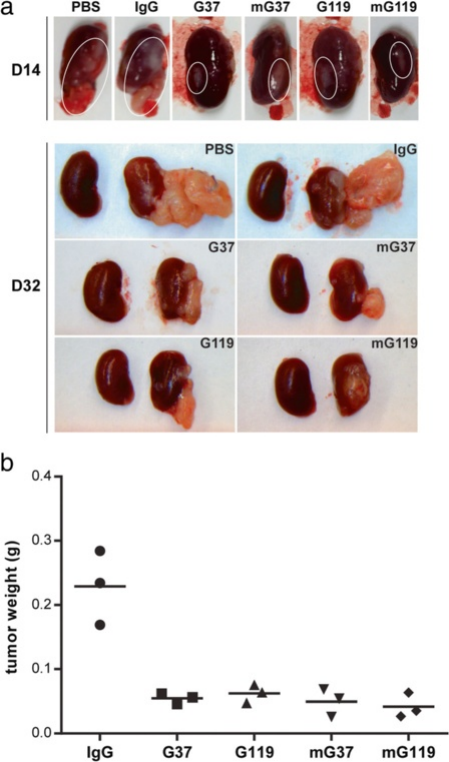
Gross pathological examination of orthotopic tumors. (**a**) Kidneys and engrafted tumors were excised from mice at the indicated time point. Upper panel, white circles indicate area of the kidney where tumors were injected into the sub-renal capsule. Lower panel, normal kidneys (left) versus kidneys bearing engrafted tumors (right) from the indicated treatment groups. All images are taken from one mouse, and are representative of the indicated treatment group. (**b**) Tumor weight was determined by excision of the engrafted tumor on day 32 from the indicated treatment group

To demonstrate the specific killing activity of fully human anti-CAIX mAbs against CAIX-expressing cells, we engineered the rapidly growing CAIX-negative RCC cell line SKRC-59 to stably express human CAIX (CAIX^+^ SKRC-59) by lentiviral transduction. Furthermore, in light of the known varied ability of healthy human PBMC to elicit ADCC responses, we first pre-selected PBMC from a single donor that exhibited the highest capacity to promote killing of RCC cells in the presence of anti-CAIX G37 *in vitro* (Additional file 2: Figure S2). Following engraftment of tumors, and injection of mice on day 4 with the human PBMC that exhibited high ADCC, and with mAbs on day 10, all groups showed a small but appreciable decrease in tumor growth beyond one week post engraftment (Fig. 5a). Through two weeks post tumor engraftment, no significant difference in tumor growth was seen between treatment groups by BLI analysis. However, at three weeks, mice treated with PBS or an irrelevant IgG1 showed an increased growth of the orthotopic tumors. In contrast, mice treated with anti-CAIX mAbs demonstrated significantly less tumor growth by BLI analysis (Fig. 5b). At day 14 post tumor engraftment (10 days after PBMC injection and 4 days after antibody injection), gross pathological examination revealed a more pronounced growth of the tumors in mice treated with control antibody and PBS than mice treated with anti-CAIX mAbs (Fig. 6a, upper panel). Gross inspection of tumors at the terminal time point (day 32) (Fig. 6a, lower panel) and measurement of tumor mass (Fig. 6b) demonstrated that control mice had substantially larger tumor burden that broke free of the subrenal capsule to appose the abdominal wall, while mice treated with anti-CAIX mAbs had tumors that remained attached to the kidney parenchyma. These findings correlate with the BLI analysis (Fig. 5b), and emphasize that the fully human anti-CAIX mAbs markedly limit tumor growth *in vivo*.

### Potential immune mechanisms of anti-CAIX mAb-mediated tumor inhibition in the allogeneic PBMC orthotopic model

**Fig. 7:**
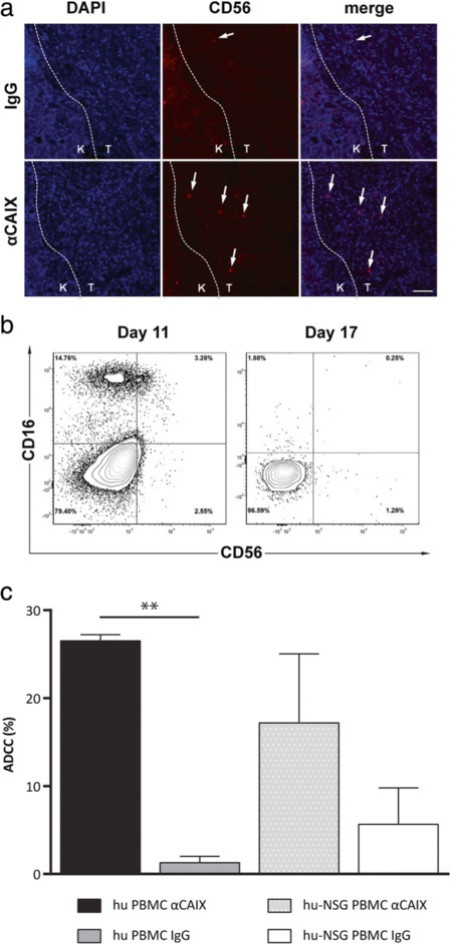
Human NK cell analysis. (**a**) Kidneys were excised from mice one day after antibody injection and examined for human NK cell influx into the engrafted tumor by immmunofluorescent analysis of human NK cells (CD56^+^, red) and contrasted against DAPI (blue). Data are from the IgG1 and G37 treatment groups, and representative of control and anti-CAIX treatments, respectively. (**b**) Human NK cell populations were analyzed by FACS after gating on human CD45^+^CD3^−^ cells in the peripheral blood of mice at day 11 and 17 from mice in the G37 treatment group, (**c**) The ADCC of freshly isolated human PBMC (left, hu-PBMC) and PBMC isolated from NSG mice 32 days after human PBMC injection from the same donor (hu-NSG PBMC, right) were determined against RCC cells used to establish orthotopic tumors with control or G37 IgG1. Data represent means of three measurements. ** indicates p value of student *t*-test < 0.01, compared to control IgG1

The NOD/SCID/IL2Rγ^−/−^ (NSG) mouse was selected as the background strain for these studies because of its lack of endogenous immune killing mechanisms by ADCC (natural killer (NK) and myeloid cells) and CDC activity [35], and its high rate of xenogenic tumor engraftment [27]. The lack of tumor growth inhibition early in treatment (Fig. 5), and significant growth of tumors only in mice not treated with anti-CAIX mAbs (Fig. 6), raised the question as to what cells might be involved in facilitating anti-CAIX mAb mediated tumor growth inhibition. We first examined NK cells in engrafted tumors on day 11 (one day after mAb injection and seven days after PBMC injection, Fig. 4b), and found that there were greater numbers of detectable human NK cells in the tumors of anti-CAIX mAb-treated mice than in control-treated mice (Fig. 7a). Although CD56 staining might also point to NK-T cells, the ratio of NK:NK-T cells in PBMCs is reported as 15:1 [36] and their contribution if any, remains to be determined. Human CD56^+^NK cells were additionally stained with CD16^+^ to detect cells that are active for ADCC [37] in the periphery of mice on day 11 (3.28 %), but they were undetectable in either the peripheral blood (Fig. 7b) or tumors (data not shown) on day 17 (0.25 %). These data indicate that while NK cells may have affected tumor growth during the first week after human PBMC injection and at least one day after mAb injection, NK cells were not detected in the blood of NSG mice at day 17 post tumor implantation, and therefore did not appear to be directly involved in the inhibition of tumor growth that was seen at later time points.

The simple explanation that the anti-tumor activity was mediated by myeloid cells still present in NSG mouse blood or by complement was also investigated. We performed *in vitro* killing studies to determine whether G37 and G119 IgG1 can induce cytotoxicity of CAIX^+^ SKRC-59 cells in the presence of non-activated NSG PBMCs (Additional file 3: Figure S3a) or NSG serum (Additional file 3: Figure S3b), mouse anti-human CAIX IgG2a serving as a positive control. As can be seen, human G119 IgG1 was not effective in inducing ADCC or CDC whereas the mouse anti-CAIX mAb was able to induce a modest level of killing, likely do to more efficient engagement of mouse FcγRIII than human IgG1 [38–41].

**Fig. 8:**
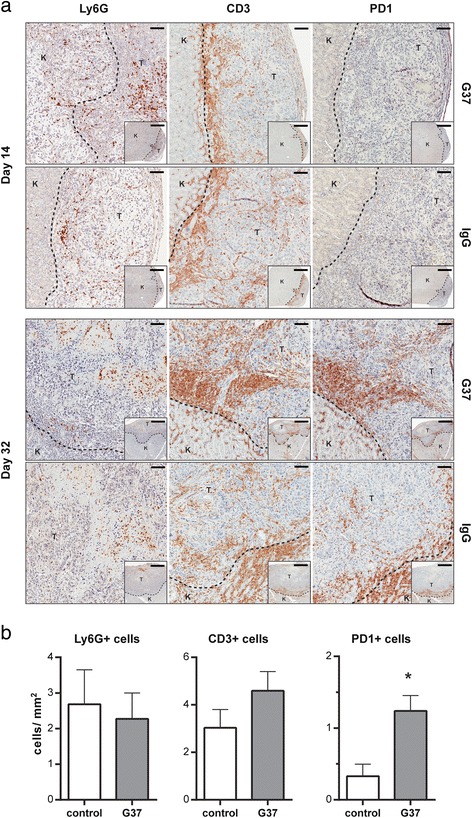
Histological analysis of immune infiltrates in orthotopic RCC tumors. (**a**) Immunohistochemical detection of Ly6G^+^, human CD3^+^, or human PD-1^+^ cells in the tumor sections at day 14 (upper panels) and day 32 (lower panels) of indicated treatment group. Large panels represent a 10x magnification of the indicated treatment group (rows) for detection of each marker (columns), with the smaller inset of 2x magnification indicating the magnified area. In each magnification, “K” indicates the mouse kidney and “T” represents the orthotopic tumor. Bars scale, 150 μm and 750 μm for 10x and 2x magnification, respectively. (**b**) The cell numbers per mm^2^ were measured in the tumor sections by Aperio and ImageScope software from at least three sections of each tumor in the indicated treatment group. * indicates p value of student *t*-test < 0.05, compared to control IgG1

Despite the loss of NK cells over time in the model and lack of ADCC by non-activated NSG myeloid cells, total PBMCs from these treated mice contained both human and mouse cells and retained ADCC activity at the terminal time point (day 32) that was similar to freshly isolated human PBMCs (Fig. 7c). We therefore examined other cells that might directly or indirectly mediate tumor inhibition in the presence of anti-CAIX mAbs. At day 14 post tumor engraftment, mouse myeloid cells (Ly6G^+^) infiltrated the site of tumor engraftment for all treatment groups (Fig. 8a, upper panel), suggesting that activated myeloid cells were being recruited. Similarly, human T cells (CD3^+^) were detectable in the tumors of all treatment groups two weeks after injection, but these T cells did not exhibit a high level of PD-1 expression, a marker of activation and exhaustion [42]. At the terminal time point (32 days after tumor engraftment), human T cell and mouse myeloid cell tumor infiltrates persisted or increased (Fig. 8a, lower panel), and this infiltration was again similar across all treatment groups. However, when we performed histological quantification of tumor infiltrates (Fig. 8b), the analysis revealed that mice treated with G37 mAb have higher levels of T cells expressing PD-1. To examine if the infiltrating T cells were activated, serial sections of the tumor tissue were stained with anti-CD8 and anti-IFN-γ antibodies. The tumor tissues of anti-CAIX mAbs treated cases consistently showed remarkable infiltration of CD8^+^ T cells in all groups (Additional file 4: Figures S4 and Additional file 5: Figure S5). Moreover, the IFN-γ-staining T cells from anti-CAIX mAbs treated tumor tissues distinctly showed more dense staining of positive cells compared to control IgG1-treated group. Together, these findings suggest that, in the presence of anti-CAIX mAbs, the tumor inhibition seen at later time points in our model may involve both activated T cells and endogenous (Ly6G^+^) mouse myeloid cells.

## Discussion

Although treatment of RCC with antibody-based therapies has increased over the last several years [5], cancer cures with this approach has remained elusive [2, 3]. Various antibodies targeting the tumor-associated antigen CAIX on RCC cells have been amongst those most extensively studied [8, 21, 23, 24, 34], owing to the antigen’s high expression on RCC. One chimeric anti-CAIX antibody that does not inhibit enzymatic activity, girentuximab, has been evaluated in the clinic [24, 43]. In preliminary analysis of Phase 3 trial results, an increase in the duration of disease-free survival was seen only in a subgroup of younger patients who exhibited high expression of CAIX on RCC tumors. Several small molecules known to effectively inhibit CA have demonstrated particular promise as potential anti-cancer agents [44, 45]. These findings highlight a need to both more clearly define patient populations under investigational drug study and develop novel anti-CAIX antibody drug candidates to increase overall response rate of patients with CAIX^+^ RCC tumors. Thus, anti-CAIX antibodies that combine inhibition of the enzymatic activity of CAIX with ADCC or CDC might be more potent than girentuximab.

In this study, we selected several of our anti-CAIX mAbs for further characterization based on their high affinity, capacity to inhibit CA activity, or ability to induce CAIX internalization (Additional file 6: Table S1) [23]. Our findings indicate that when CAIX-expressing RCC cells were simultaneously incubated with internalizing or non-internalizing anti-CAIX mAbs and PBMCs, ADCC activity of the antibodies showed no correlation with their reported capacity to induce CAIX internalization (Fig. 1). Similarly, the capacity to induce CAIX internalization did not affect the ability of anti-CAIX mAbs to limit RCC motility, as both G37 (non-internalizing) and G119 (internalizing) IgG1 demonstrated comparable inhibition of RCC expansion and movement *in vitro* (Fig. 2). In addition, Fc engineering to enhance effector activity [31] increased the ADCC mediated killing of RCC by both mAbs (Fig. 3a), but had only marginal effects on CDC and phagocytosis (Figs. 3b and c). This enhanced *in vitro* ADCC killing activity did not translate into more potent inhibition of tumor growth *in vivo*. However, this may have occurred because of the high doses (10 mg/kg) and frequent treatments (2x/week) of the anti-CAIX mAbs. Additional dose response studies on the wild type and Fc mutated mAbs should be performed in future studies to determine if an enhancement in potency of tumor killing activity by Fc engineered antibodies could be seen at lower concentrations *in vivo*.

Animal models examining the efficacy of cancer therapeutics increasingly use a xenograft approach where cancer cell lines or patient derived tumor tissue are implanted into immunodeficient mice [27]. These patient-derived tumor models (PDTM) have been used in several studies of RCC [33, 34, 46, 47], and offer a promising means by which targeted therapeutics such as tyrosine kinase inhibitors can be evaluated. A caveat to this approach, however, is that the complexities of tumor and immune cell interactions are not evaluated in current RCC PDTM, and therefore these critical parameters are often missing or underrepresented in the analysis [33]. This shortcoming is mirrored in the analysis of antibody therapeutics, most immediately because of a lack of a functional immune system in mice engrafted with human tumors. We sought to circumvent this by developing an allogeneic mouse model whereby the capacity of antibody therapies against CAIX can be evaluated (Fig. 4). To this end, we created a model wherein human donor PBMC with a demonstrated capacity to kill CAIX^+^ RCC cells by ADCC (Additional file 2: Figure S2) were injected into mice bearing an orthotopic RCC tumor. To characterize the tumor killing activity mediated by anti-CAIX mAbs, a luciferase-expressing CAIX^+^ RCC cell line was transplanted orthotopically to facilitate measurement of tumor growth. We anticipated that these efforts would afford us the capacity to examine the efficacy of anti-CAIX mAbs in the presence of human immune cells to inhibit tumor growth *in vivo*.

While our mouse model demonstrated that both wild type and mutant Fc formats of anti-CAIX G37 and G119 IgG1 were capable of limiting tumor growth, this observation did not manifest by BLI until circa three weeks after PBMC and anti-CAIX treatment (Fig. 6). A possible reason for the delay in detecting tumor inhibition may be the limitations of BLI sensitivity in measuring small orthotopic tumors. Alternatively, tumor inhibition may have been delayed because several subsets of immune cells in human PBMCs do not persist in the NSG mouse for long periods of time.

Consistent with previous reports [48, 49], we were unable to detect human NK cells in the mice 13 days after PBMC injection (Fig. 7b). However, we did find that a greater influx of human NK cells occurred in tumors one day after antibody injection (7 days after PBMC injection) in mice treated with anti-CAIX mAbs (Fig. 7a). This indicates that, while human NK cells do not persist, these cells likely contributed to early inhibition of tumor growth by the anti-CAIX mAbs, which did not manifest overtly until much later time points.

An additional possibility for the inhibition of tumor growth seen in anti-CAIX mAb-treated mice may involve the influence and interaction of human T cells and mouse myeloid cells. Human T cells infiltrating the engrafted tumor appear most activated at later time points in our model, as measured by expression of human PD-1 and IFN-γ [42] (Fig. 8 and Additional file 4: Figure S4). This activation of human T cells is coincident with mouse myeloid influx in the tumor. While the myeloid compartment in NSG mice has been characterized as deficient [50], it is possible that shed CAIX/anti-CAIX complexes can active the mouse myeloid compartment and through direct cellular interactions and cytokine secretion lead to further activation of human T cells to express PD-1 (Fig. 8b) [51]. It is also possible that graft versus host disease [52] or HLA-mismatch with the engrafted tumor can act in concert with anti-CAIX mAbs and mouse myeloid cells to enhance CAIX^+^ tumor cell killing. It should also be noted that the SKRC-59 cells used for the *in vivo* studies herein are CAIX^+^PD-L1^+^ (data not shown) and therefore the potential role that the PD1:PD-L1 checkpoint control axis may play in limiting the Teff response must also be considered.

Finally, PBMC from the terminal time point (including both human immune and mouse myeloid cells) demonstrate ADCC activity, despite lacking human NK cells, which suggests that enhancement of NSG myeloid cell activity may have occurred (Fig. 7c). While our data demonstrates a lack of CDC activity *in vivo* (Additional file 3: Figure S3b) owing to a 2-base pair deletion in the complement C5 structural gene [53], it is possible that anti-CAIX deposition on the tumor cells can lead to activation of earlier complement components including C3a and C4a that can lead to chemotactic recruitment of myeloid cells and C3b to promote opsonization. Both of these actions could lead to myeloid cell activation and enhanced tumor killing activity [54]. This speculation clearly requires more extensive characterization of infiltrating human T cell and mouse myeloid cell activity in the tumor. Therapeutic antibodies targeting the PD-1:PD-L1 axis [55, 56] in combination with anti-CAIX mAbs would be particularly amenable to assessment with our model as PD-L1 is known to be expressed on RCC cells and associated with poor outcome [57].

## Conclusions

Recent clinical trials [26] highlight the pressing need for the development of novel therapeutics targeting CAIX on RCC and other cancers [16, 24]. This work demonstrates that, in addition to previously described characteristics [23], several of our high affinity, fully human anti-CAIX mAbs mediate potent ADCC and are efficient in eliciting immune mediated killing of RCC. Our development of a novel orthotopic RCC model utilizing allogeneic human PBMC confirms the capacity of these mAbs to mediate tumor growth inhibition *in vivo*. Our study provides promising new data that supports further clinical development of these anti-CAIX mAbs, particularly in regards to patients that are poised to most immediately benefit from CAIX-targeting therapeutics [5, 26] in RCC. In addition, the recently defined role of CAIX in the incidence and progression of breast cancer [58–61] offers another potential means by which the dual-targeting capacity of these anti-CAIX mAbs may be leveraged.

## Materials and methods

### Cell lines and culture

Human renal clear cell carcinoma (RCC) cell lines, SKRC-09 (CAIX positive), SKRC-52 (CAIX positive), and SKRC-59 (CAIX negative), were obtained from Dr. Gerd Ritter (Memorial Sloan-Kettering Cancer Center, New York, NY). These lines were grown in RPMI 1640 Medium (Life Technologies) supplemented with 10 % (v/v) heat-inactivated fetal bovine serum (FBS, Gibco), 100 IU/ml penicillin and 100 μg/ml streptomycin (complete medium) at 37 °C with 5 % CO_2_. RCC4 and 293 T cells (CRL-11268, ATCC) were grown in DMEM Medium (Life Technologies) supplemented with 10 % FBS, 100 IU/ml penicillin and 100 μg/ml streptomycin. The 293 F cell line (Life Technologies) was propagated and transfections for protein production carried out in 293 FreeStyle serum-free medium (Life Technologies).

### Antibodies and flow cytometry analysis

Anti-CAIX antibody variants (G10, G36, G37, G39, and G119) and control antibody were produced as described previously [23, 30]. Briefly, scFv-Fcs were constructed by cloning the single-chain variable region (scFv) in frame with a human IgG1 Fc region lacking a CH1 domain. Full length, human IgG1s were generated by cloning heavy chain variable region (VH) and light chain variable region (VL) into a dual-promoter expression cassette [4]. Antibodies (scFv-Fc and IgG1) were transfected into 293 F cells using polyethylenimine and purified by protein A sepharose affinity chromatography via ÄKTAPurifier FPLC (GE Healthcare). To characterize nature killer cells, cells were stained with human CD45, CD56 and CD16 antibodies (BioLegend) and analyzed by flow cytometry.

### Antibody-dependent cell-mediated cytotoxicity assay

Cytotoxicity assays utilized measurement of lactose dehydrogenase (LDH) release, with PBMC from healthy human donors or NSG mice used as effector cells. RCC cell lines SKRC-09, SKRC-52, SKRC-59, or CAIX^+^ SKRC-59 (engineered to express CAIX by lentiviral transduction [23]) were used as target cells and plated at 1 × 10^4^/well in a 96-well plate. Cells were incubated with indicated concentrations of antibody for one hour, followed by co-culture with effector cells for 4–6 h [4]. Culture supernatants were harvested by centrifugation and LDH measured in the supernatant by colorimetric assay (Promega) at 490 nm. The percent cytotoxicity was calculated as: %Cytotoxicity = 100 × (E – SE – ST)/(M – ST); E, released LDH from E/T culture with antibody; SE, spontaneous released LDH from effectors; ST, spontaneous released LDH from targets; M, the maximum released LDH from lysed targets. All tests were performed in triplicate.

### Complement dependent cytotoxicity assay

SKRC-52 cells were cultured in 96 well plates (2 × 10^4^/well) RPMI 1640 Medium (Life Technologies), supplemented with 100 IU/ml penicillin and 100 μg/ml streptomycin, and rabbit serum (Cedarlane Laboratories). Control or CAIX antibodies were added at the indicated amounts and cytotoxicity measured as in ADCC assays by LDH release after 6 h. Cytotoxicity was calculated as: %Cytotoxicity = 100 × (E – ST)/(M – ST). All tests were performed in triplicate.

### Migration assay

Migration of SKRC-52 cells was determined by transwell assay using FluoroBlok 24-multiwell insert assay (BD Biosciences). Overnight starved (0.5 % FBS) SKRC-52 cells were labeled with a lipophilic fluorescent dye DiO (Life Technology) and seeded in the upper chamber at 1 × 10^5^ cells/well in 0.5 % FBS RPMI in the presence or absence of 2.5 μg/ml G37, G119, a non-specific IgG1, or 100 μM of acetazolamide as a positive control for inhibition of migration. Hepatocyte growth factor (HGF, Sigma) was added into the lower chamber. Fluorescence intensity of the lower chamber, indicating cell migration, was measured after 24-h incubation at 37 °C using a POLARstar reader (BMG Labtech). All tests were performed in triplicate.

### Wound healing assay

SKRC-52 cells were cultured to confluency in 24-well plates (approximately 2 × 10^5^ cells/well). The monolayers were incubated in the absence of serum for 16 h and wounded in a line across the well with a 200-μl pipette tip. The wounded monolayers were washed twice with serum-free media and incubated with 10 μg/ml of G37, G119, a non-specific IgG1, or 100 μM acetazolamide. The area of cell-free wound was recorded 24-h later using a charge-coupled device camera (C2400; NEC) on an inverted microscope (Axiovert 35; Zeiss) and analyzed with image analysis software (NIH Image 1.55). Wound healing was calculated as the percentage of the remaining cell-free area compared with the area of the initial wound. All tests were performed in triplicate.

### Cell growth inhibition assay

SKRC-52 cells were seeded in 96-well plates at a density of 4 × 10^3^ cells/well. After 24 h, cells were incubated with anti-CAIX antibodies or control antibody in fresh medium. Followed by 24, 48, 72, 96, and 120 h incubation, MTT (2 mg/ml in RPMI, 50 μl/well) was added to the wells and further incubated at 37 °C for 2.5 h. The supernatant was removed and 150 μl DMSO per well was added to dissolve the produced formazan. After shaking the plates for 10 min, optical absorbance at 570 nm were recorded with a microplate reader.

### Antibody dependent cellular phagocytosis assay

CD14 positive monocytes were isolated from healthy donor whole blood by fluorescent activated cell sorting (FACS), and cultured in 10 cm^2^ dishes with complete medium supplemented with 200 U/ml granulocyte macrophage colony-stimulating factor (GM-CSF, eBioscience). Culture medium was refreshed every other day for 5 days, non-adherent cells were washed away with PBS and the adherent monocyte-derived macrophages (MDMs) harvested. SKRC-52 cells were labeled with PKH-26 fluorescent membrane dye (Sigma), washed three times, incubated on ice for 1 h without antibody or CAIX antibodies, and cultured with MDMs at a 1:8 ratio for 4 h at 37 °C. Phagocytosis was determined after extensive washing of the co-culture, and measured by FACS as CD14^+^PKH^+^ cells. The percentage of phagocytosis was calculated as: %Phagocytosis = 100 × [(percent dual positive)/(percent dual positive + percent residual targets)]. All tests were performed in triplicate.

### Orthotopic RCC tumor: allogeneic human PBMC model

Orthotopic RCC tumors were established by injecting 5 × 10^4^ SKRC-59 cells into the left subrenal capsule of kidneys of NSG mice. SKRC-59 cells were engineered to express high levels of human CAIX and luciferase, through lentiviral transduction, and passaged subcutaneously in NSG mice prior to engraftment to enrich for rapidly growing, CAIX^+^ cells (data not shown). Prior to injection, RCC cells were suspended in PBS and diluted 1:1 in Matrigel (Life Technologies) to ensure retention of the cells within the subrenal capsule. Three days after engraftment, tumor implantation was confirmed by bioluminescence imaging (BLI) using a Xenogen imaging system (Life Technology) with subsequent measurements taken every 3–4 days thereafter. Four days after tumor engraftment, 1×10^7^ PBMC from a healthy human donor (selected for high ADCC activity) were injected intravenously. Six days after PBMC injection (10 days after engraftment) control or CAIX antibodies were injected at 10 mg/kg intravenously, and subsequently injected every 3–4 days thereafter. Mice were sacrificed and tissues harvested at 32 days post tumor engraftment. Animal care was carried out in accordance with the guidelines of Animal Care and Use Committee of Dana-Farber Cancer Institute.

### Histology

RCC tumors and kidneys were surgically removed, fixed in 10 % neutral buffered formalin, embedded in paraffin, and 6-μm thick sections placed on slides. Hemotoxylin and eosin (H&E) immunostaining was performed using anti-human CD3 (A0452, DAKO), anti-human PD-1 (315 M–96, Cell Marque), anti-mouse Ly6G (1A8, BioLegend), anti-IFN-γ (MD-1, BioLegend), and anti-CD8 (M7103, DAKO) antibodies. HRP-labeled anti-rabbit antibodies (Pierce) and Mouse on Mouse (M.O.M.) Kits (Vector) were used as secondary antibodies and detected by diaminobenzidine according to the manufacturer’s instructions. Imaging of sectioned tissues was carried out using the ScanScope system (Aperio) and the ImageScope software at the Dana-Farber/Harvard Cancer Center Tissue Microarray and Imaging Core. Tumor sections for immunofluorescent staining was performed as above, but with 4’,6-diamidino-2-phenylindole (DAPI, Life Technologies) and phycoerythrin (PE) mouse anti-human CD56 (MEM188, BioLegend). Fluorescent images were acquired on a Leica confocal microscope (TCS-SP5-AOBS) and images overlaid using Leica application suite advanced fluorescence software.

### Statistical analysis

Data was analyzed using two-sided unpaired Student’s *t*-test or two-way ANOVA. *, **, and *** indicate p < 0.05, 0.01 and 0.005, respectively. All values and bars are represented as mean +/− standard deviation (S.D.) or standard error of the mean (S.E.M.).

## Additional files

Additional file 1: Figure S1. The antibody-mediated killing activities on CAIX expressing RCC4 tumor cells. (a) Anti-CAIX antibodies (G37 and G119) were tested for ADCC activity with human PBMCs in a dose dependent manner. CAIX+ RCC4 cells were incubated with human PBMCs at an indicated ratio and 5 μg/ml of antibodies, and cytotoxicity measured by LDH release in the culture supernatant. Data represent triplicate wells of one experiment. (b) CDC activity was determined by culture of rabbit serum with RCC4 cells, and cytotoxicity measured as above after 6 h. All data points represent the mean value ± S.D. *, **, and *** represent p value of Student *t*-test < 0.05, 0.01, and 0.005, respectively. Additional file 2: Figure S2. Selection of human PBMC with high ADCC activity. Human PBMC, isolated from twenty-one healthy donors were cultured with CAIX^+^ SKRC-59 cells (25:1 PBMC: RCC) in the presence of the indicated concentration of anti-CAIX G37. ADCC activity was measured as described in Materials and Methods, with donor 7 (D7) was chosen as the source of human PBMC utilized in the *in vivo* mouse model construction. Data represent the mean of triplicate measurements, ± S.D. Additional file 3: Figure S3. The antibody-mediated killing activities on CAIX expressing tumor cells through NSG blood or serum. (a) Human anti-CAIX mAbs (G37 or G119) and mouse antihuman CAIX antibody, MAB2188, were tested for ADCC activity against CAIX+ SKRC-59 cells. NSG mouse PBMCs were isolated from untreated NSG mice, and total PBMC counted as effector cells (E) in co-culture with the respective target CAIX+ SKRC-59 cells (T) for six hours. The concentration of antibodies was 5 μg/ml. (b) CDC activity was determined by culture of NSG mouse serum with CAIX+ SKRC-59 cells, and cytotoxicity measured as above after 6 h. Culture supernatant was examined for LDH as a measure of cytotoxicity. Data represent the mean of three independent experimental values, ± S.D.. * represents p value of Student *t*-test < 0.05. Additional file 4: Figure S4. IFN-γ and CD8^+^ T cells staining on orthotopic RCC tissues. Representative immunohistochemical staining for IFN-γ (upper two lanes) and CD8 (lower lane) in the tumor sections at day 32 was shown by the indicated treatment group. Anti-IFN-γ and anti-CD8 antibodies were detected by DAB (shown as brown particles) and indicated by arrows. Bars represent 50 μm. Additional file 5: Figure S5. IHC staining for HRP-labeled secondary anti-rabbit antibody and mouse on mouse (M.O.M.) kit. Representative immunohistochemical staining for HRP-labeled anti-rabbit antibody (upper) and M.O.M. kit (lower) in the tumor sections at day 32 was shown by the G37 treatment group. Sections were further detected by DAB. Bars represent 100 and 50 μm in 40X and 20X, respectively. Additional file 6: Table S1. The kinetic constants of all anti-CAIX antibodies were obtained by global analysis using a 1:1 langmuir binding model and the T100 evaluation software. Percentage of carbonic anhydrase inhibition by CAIX antibodies was estimated from an electrometric assay. Internalization measured by both flow cytometry and ImageStream. + denotes degree of internalization as measured by regular flow cytometry.
