# Supplementary material for: Human anti-CAIX antibodies mediate immune cell inhibition of renal cell carcinoma in vitro and in a humanized mouse model in vivo
Source: Mol Cancer. 2015 Jun 11;14:119. doi: 10.1186/s12943-015-0384-3 (PMC4464115; doi:10.1186/s12943-015-0384-3)
Supplement: Additional file 1: Figure S1. — The antibody-mediated killing activities on CAIX expressing RCC4 tumor cells. (a) Anti-CAIX antibodies (G37 and G119) were tested for ADCC activity with human PBMCs in a dose dependent manner. CAIX+ RCC4 cells were incubated with human PBMCs at an indicated ratio and 5 μg/ml of antibodies, and cytotoxicity measured by LDH release in the culture supernatant. Data represent triplicate wells of one experiment. (b) CDC activity was determined by culture of rabbit serum with RCC4 cells, and cytotoxicity measured as above after 6 h. All data points represent the mean value ± S.D. *, **, and *** represent p value of Student t-test < 0.05, 0.01, and 0.005, respectively. [file 12943_2015_384_MOESM1_ESM.pdf]

Supplementary Figure 1

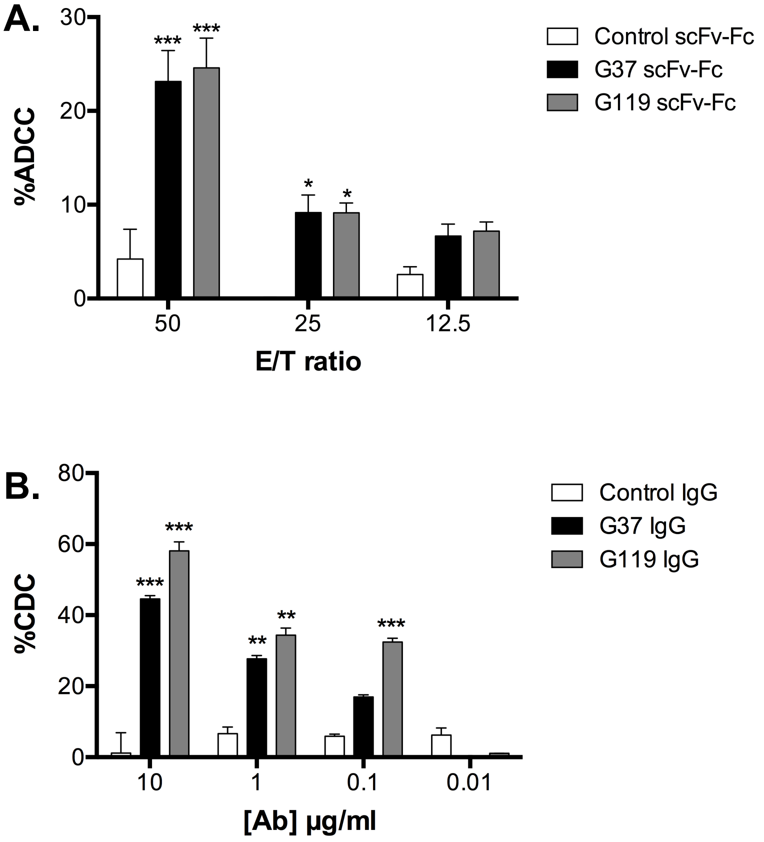

Figure S1. The antibody-mediated killing activities on CAIX expressing RCC4 tumor cells. **(A)** Anti-CAIX antibodies (G37 and G119) were tested for ADCC activity with human PBMCs in a dose dependent manner. $CAIX^+$ RCC4 cells were incubated with human PBMCs at an indicated ratio and 5 μg/ml of antibodies, and cytotoxicity measured by LDH release in the culture supernatant. Data represent triplicate wells of one experiment. **(B)** CDC activity was determined by culture of rabbit serum with RCC4 cells, and cytotoxicity measured as above after 6 hours. All data points represent the mean value ±S.D.. *, **, and *** represent p value of Student t-test < 0.05, 0.01, and 0.005, respectively.
