# Supplementary material for: Human anti-CAIX antibodies mediate immune cell inhibition of renal cell carcinoma in vitro and in a humanized mouse model in vivo
Source: Mol Cancer. 2015 Jun 11;14:119. doi: 10.1186/s12943-015-0384-3 (PMC4464115; doi:10.1186/s12943-015-0384-3)
Supplement: Additional file 2: Figure S2. — Selection of human PBMC with high ADCC activity. Human PBMC, isolated from twenty-one healthy donors were cultured with CAIX+ SKRC-59 cells (25:1 PBMC: RCC) in the presence of the indicated concentration of anti-CAIX G37. ADCC activity was measured as described in Materials and Methods, with donor 7 (D7) was chosen as the source of human PBMC utilized in the in vivo mouse model construction. Data represent the mean of triplicate measurements, ± S.D. [file 12943_2015_384_MOESM2_ESM.docx]

**Supplementary Figure 2**

**
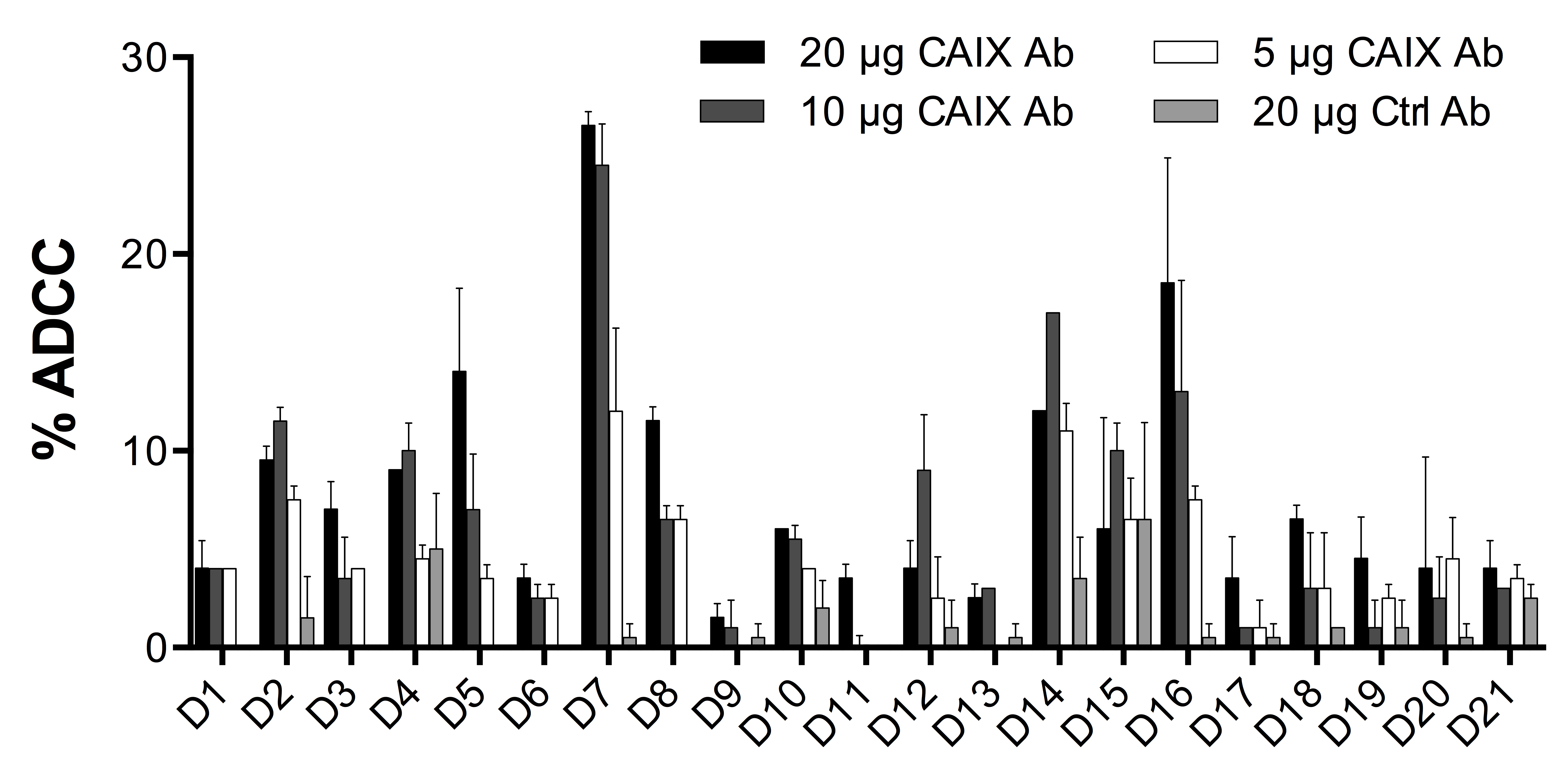
**

**Figure S2. Selection of human PBMC with high ADCC activity.** Human PBMC, isolated from twenty-one healthy donors were cultured with CAIX^+^ SKRC-59 cells (25:1 PBMC:RCC) in the presence of the indicated concentration of anti-CAIX G37. ADCC activity was measured as described in Materials and Methods, with donor 7 (D7) was chosen as the source of human PBMC utilized in the *in vivo* mouse model construction. Data represent the mean of triplicate measurements, ± S.D..
