# Supplementary material for: Human anti-CAIX antibodies mediate immune cell inhibition of renal cell carcinoma in vitro and in a humanized mouse model in vivo
Source: Mol Cancer. 2015 Jun 11;14:119. doi: 10.1186/s12943-015-0384-3 (PMC4464115; doi:10.1186/s12943-015-0384-3)
Supplement: Additional file 3: Figure S3. — The antibody-mediated killing activities on CAIX expressing tumor cells through NSG blood or serum. (a) Human anti-CAIX mAbs (G37 or G119) and mouse antihuman CAIX antibody, MAB2188, were tested for ADCC activity against CAIX+ SKRC-59 cells. NSG mouse PBMCs were isolated from untreated NSG mice, and total PBMC counted as effector cells (E) in co-culture with the respective target CAIX+ SKRC-59 cells (T) for six hours. The concentration of antibodies was 5 μg/ml. (b) CDC activity was determined by culture of NSG mouse serum with CAIX+ SKRC-59 cells, and cytotoxicity measured as above after 6 h. Culture supernatant was examined for LDH as a measure of cytotoxicity. Data represent the mean of three independent experimental values, ± S.D.. * represents p value of Student t-test < 0.05. [file 12943_2015_384_MOESM3_ESM.pdf]

Supplementary Figure 3

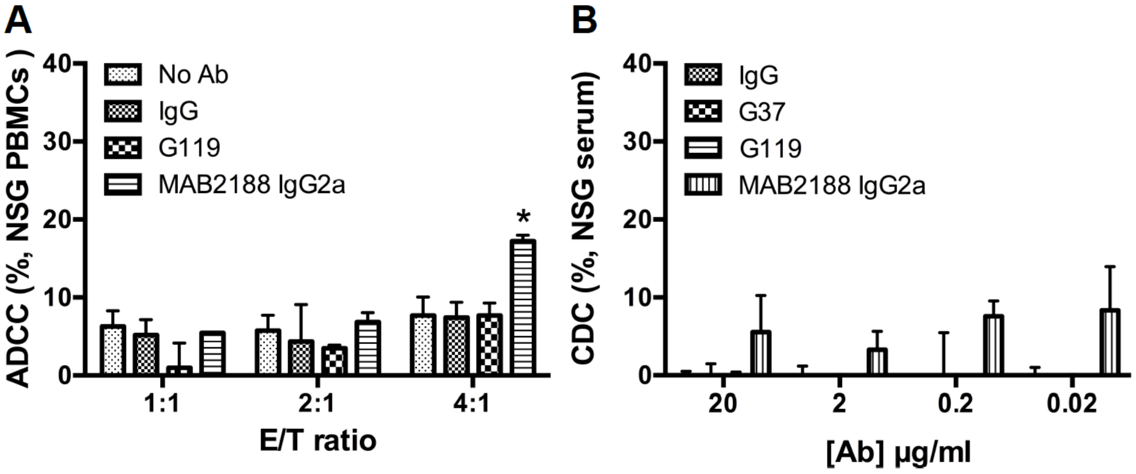

Figure S3. The antibody-mediated killing activities on CAIX expressing tumor cells through NSG blood or serum. (A) Human anti-CAIX mAbs (G37 or G119) and mouse anti-human CAIX antibody, MAB2188, were tested for ADCC activity against CAIX$^+$ SKRC-59 cells. NSG mouse PBMCs were isolated from untreated NSG mice, and total PBMC counted as effector cells (E) in co-culture with the respective target CAIX$^+$ SKRC-59 cells (T) for six hours. The concentration of antibodies was 5 μg/ml. **(B)** CDC activity was determined by culture of NSG mouse serum with CAIX$^+$ SKRC-59 cells, and cytotoxicity measured as above after 6 hours. Culture supernatant was examined for LDH as a measure of cytotoxicity. Data represent the mean of three independent experimental values, ± S.D.. * represents p value of Student t-test < 0.05.
