# Supplementary material for: Human anti-CAIX antibodies mediate immune cell inhibition of renal cell carcinoma in vitro and in a humanized mouse model in vivo
Source: Mol Cancer. 2015 Jun 11;14:119. doi: 10.1186/s12943-015-0384-3 (PMC4464115; doi:10.1186/s12943-015-0384-3)
Supplement: Additional file 4: Figure S4. — IFN-γ and CD8+ T cells staining on orthotopic RCC tissues. Representative immunohistochemical staining for IFN-γ (upper two lanes) and CD8 (lower lane) in the tumor sections at day 32 was shown by the indicated treatment group. Anti-IFN-γ and anti-CD8 antibodies were detected by DAB (shown as brown particles) and indicated by arrows. Bars represent 50 μm. [file 12943_2015_384_MOESM4_ESM.docx]

**Supplementary Figure 4**

**
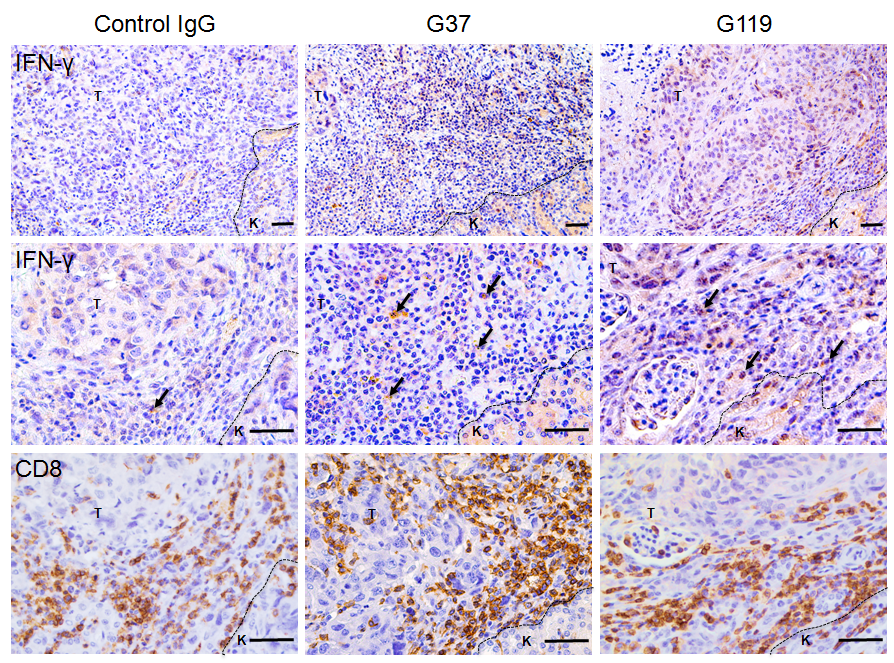
**

**Figure S4. IFN-γ and CD8^+^ T cells staining on orthotopic RCC tissues.** Representative immunohistochemical staining for IFN-γ (upper two lanes) and CD8 (lower lane) in the tumor sections at day 32 was shown by the indicated treatment group. Anti-IFN-γ and anti-CD8 antibodies were detected by DAB (shown as brown particles) and indicated by arrows. Bars represent 50 µm.
