# Supplementary material for: Human anti-CAIX antibodies mediate immune cell inhibition of renal cell carcinoma in vitro and in a humanized mouse model in vivo
Source: Mol Cancer. 2015 Jun 11;14:119. doi: 10.1186/s12943-015-0384-3 (PMC4464115; doi:10.1186/s12943-015-0384-3)
Supplement: Additional file 5: Figure S5. — IHC staining for HRP-labeled secondary anti-rabbit antibody and mouse on mouse (M.O.M.) kit. Representative immunohistochemical staining for HRP-labeled anti-rabbit antibody (upper) and M.O.M. kit (lower) in the tumor sections at day 32 was shown by the G37 treatment group. Sections were further detected by DAB. Bars represent 100 and 50 μm in 40X and 20X, respectively. [file 12943_2015_384_MOESM5_ESM.docx]

**Supplementary Figure 5**

**
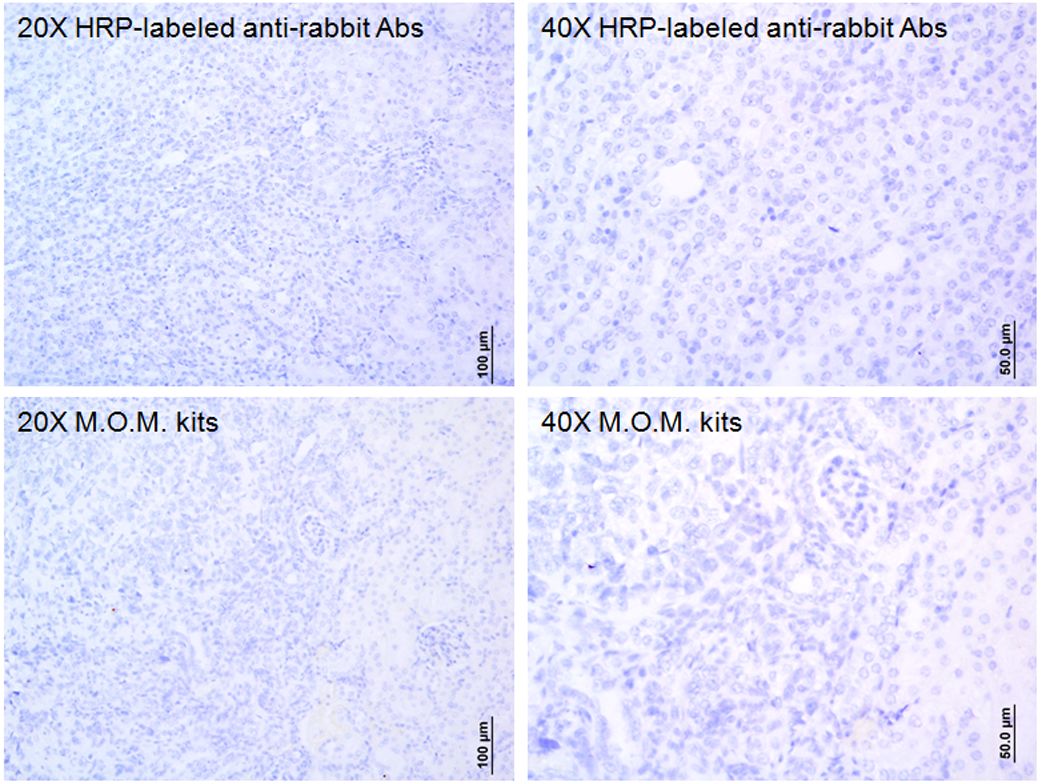
**

**Figure S5. IHC staining for HRP-labeled secondary anti-rabbit antibody and mouse on mouse (M.O.M.) kit.** Representative immunohistochemical staining for HRP-labeled anti-rabbit antibody (upper) and M.O.M. kit (lower) in the tumor sections at day 32 was shown by the G37 treatment group. Sections were further detected by DAB. Bars represent 100 and 50 µm in 40X and 20X, respectively.
