# Supplementary material for: Human anti-CAIX antibodies mediate immune cell inhibition of renal cell carcinoma in vitro and in a humanized mouse model in vivo
Source: Mol Cancer. 2015 Jun 11;14:119. doi: 10.1186/s12943-015-0384-3 (PMC4464115; doi:10.1186/s12943-015-0384-3)
Supplement: Additional file 6: Table S1. — The kinetic constants of all anti-CAIX antibodies were obtained by global analysis using a 1:1 langmuir binding model and the T100 evaluation software. Percentage of carbonic anhydrase inhibition by CAIX antibodies was estimated from an electrometric assay. Internalization measured by both flow cytometry and ImageStream. + denotes degree of internalization as measured by regular flow cytometry. [file 12943_2015_384_MOESM6_ESM.pdf]

Supplementary Table 1

αCAIX antibody	Affinity ($K_{D,}$ nM)	% CA inhibition	Internalization
G119	1.49	25%	+++
G10	1.62	15%	+
G37	1.89	40%	none
G36	3.22	10%	++
G39	3.43	50%	none

The kinetic constants of all anti-CAIX antibodies were obtained by global analysis using a 1:1 langmuir binding model and the T100 evaluation software. Percentage of carbonic anhydrase inhibition by CAIX antibodies was estimated from an electrometric assay. Internalization measured by both flow cytometry and ImageStream. + denotes degree of internalization as measured by regular flow cytometry.
